# Highly Active Antiretroviral Therapies Are Effective against HIV-1 Cell-to-Cell Transmission

**DOI:** 10.1371/journal.ppat.1003982

**Published:** 2014-02-27

**Authors:** Luis M. Agosto, Peng Zhong, James Munro, Walther Mothes

**Affiliations:** Department of Microbial Pathogenesis, Yale University School of Medicine, New Haven, Connecticut, United States of America; Vanderbilt University School of Medicine, United States of America

## Abstract

HIV-1 cell-to-cell transmission allows for 2–3 orders of magnitude more efficient viral spread than cell-free dissemination. The high local multiplicity of infection (MOI) observed at cell-cell contact sites may lower the efficacy of antiretroviral therapies (ART). Here we test the efficacy of commonly used antiretroviral inhibitors against cell-to-cell and cell-free HIV-1 transmission. We demonstrate that, while some nucleoside-analog reverse transcriptase inhibitors (NRTI) are less effective against HIV-1 cell-to-cell transmission, most non-nucleoside-analog reverse transcriptase inhibitors (NNRTI), entry inhibitors and protease inhibitors remain highly effective. Moreover, poor NRTIs become highly effective when applied in combinations explaining the effectiveness of ART in clinical settings. Investigating the underlying mechanism, we observe a strict correlation between the ability of individual drugs and combinations of drugs to interfere with HIV-1 cell-to-cell transmission, and their effectiveness against high viral MOIs. Our results suggest that the ability to suppress high viral MOI is a feature of effective ART regimens and this parameter should be considered when designing novel antiviral therapies.

## Author Summary

HIV-1 cell-to-cell transmission has gained interest due to its potential role in AIDS pathogenesis. It has recently been suggested that antiretroviral therapies fail during cell-to-cell transmission because of the high number of particles transferred at sites of cell-cell contacts. However, these findings stand in contrast with the clinical observation that ART is successful in suppressing retroviral replication in HIV-positive patients. Consequently, many interpreted this observation to suggest that HIV-1 cell-to-cell transmission is not clinically relevant. Here we show that this interpretation is likely incorrect. By systematically testing the efficacy of commonly used antiretroviral inhibitors against cell-to-cell and cell-free HIV-1 transmission, we demonstrate that, while some NRTIs are less effective, most NNRTIs, entry inhibitors and protease inhibitors remain highly effective. Moreover, NRTIs become highly effective when combined, thus supporting the known effectiveness of HAART in clinical settings. Interestingly, the ability of individual drugs and combinations to interfere with HIV-1 cell-to-cell transmission correlates with their effectiveness against high viral MOIs. Our results suggest that the ability to suppress the high viral MOI during HIV-1 cell-to-cell transmission is a critical feature of existing ART regimens that should be tested when designing novel antiviral therapies.

## Introduction

Highly active antiretroviral therapy (HAART) has significantly reduced the mortality rate and has increased the life span of HIV-infected patients by maintaining viral loads below detection levels, thus preventing the onset of AIDS [1], [2], [3], [4]. However, the presence of a stable latent reservoir, poor treatment adherence, and the emergence of drug-resistant HIV-1 variants continue to present challenges for successful treatments [5]. In order to develop more effective therapies, a detailed understanding of the pathogenesis of HIV-1 is necessary. Cell-to-cell transmission of HIV-1 has attracted significant attention as a potential factor influencing the pathogenesis of HIV-1 [6], [7].

HIV-1 cell-to-cell transmission describes efficient virus spreading via sites of cell-cell contact through formation of virological or infectious synapses [7], [8]. It provides for 2–3 orders of magnitude more efficient spread than cell-free virus dissemination and it is believed to be the main mode of viral spread *in vitro* [9], [10], [11], [12]. The formation of virological synapses allows the coordination of viral assembly with viral entry at sites of cell-cell contacts [13], [14], [15], [16], [17]. These supramolecular structures permit the efficient transfer of large numbers of infectious particles to target cells resulting in a higher viral MOI than cell-free infection [18], [19], [20], consistent with some *in vivo* observations [21], [22]. This transfer of high viral MOI can also result in bystander death of CD4+ lymphocytes [23]. Primary cells may undergo pyroptosis and/or apoptosis in response to a high load of viral DNA in the cytoplasm and/or multiple viral integration events in the nucleus [24], [25], [26]. The cell death of highly infected cells may result in the positive selection of CD4+ T cells that carry a single provirus [27], [28]. HIV-1 cell-to-cell transmission also allows HIV-1 to overcome barriers to infection and protects it from immunological and cellular restriction factors [11], [20], [29], [30], [31]. Finally, it has recently been reported that cell-to-cell transmission may protect HIV-1 from inhibition by antiretroviral therapies [32]. The transfer of large numbers of particles is thought to reduce the effective concentration of antiretroviral drugs within the cell and thus may provide a mechanism for the spread of HIV-1 in the presence of such therapies [32], [33]. A reduced effectiveness of drugs during HIV-1 cell-to-cell transmission has been reported for tenofovir (TFV), efavirenz (EFV) and zidovudine (AZT) [32], [33], [34]. However, these reports would seem to be in conflict with the clinical observation that HAART is successful at suppressing retroviral replication in millions of AIDS patients.

In this study, we tested a panel of antiretroviral drugs that include nucleoside analog reverse transcriptase inhibitors (NRTI), non-nucleoside analog reverse transcriptase inhibitors (NNRTI), entry inhibitors (Ent-I) and protease inhibitors (PI) for their ability to inhibit HIV-1 cell-to-cell transmission. We found that while some NRTI drugs lost activity when virus was transferred by cell-to-cell transmission, NNRTIs, Ent-Is and PIs remained highly effective. Importantly, we regained potent antiretroviral activity upon combining NRTIs that were ineffective towards HIV-1 cell-to-cell transmission as single therapies. These results explain the effectiveness of antiretroviral combination therapies in clinical settings. Finally, we demonstrate that the effectiveness of ART against HIV-1 cell-to-cell transmission can be recapitulated by testing their effectiveness against high viral MOI. Altogether, our results suggest that the ability to suppress high viral MOI is a defining feature of effective ART regimens and provides a valuable tool to develop novel ART that remain effective against HIV-1 cell-to-cell transmission.

## Results

**Figure 1:**
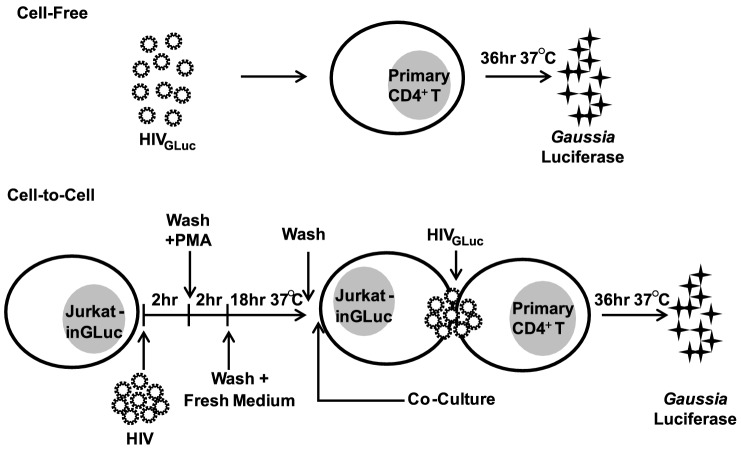
Experimental design for comparing cell-free to cell-to-cell transmission of HIV-1. (**A**) To measure cell-free HIV-1 infections, we generated HIV-1_GLuc_ by transfecting plasmids encoding for HIV-1_NL4-3_ as well as the HIV-1_inGLuc_ reporter plasmids into HEK293 cells and infected primary CD4+ T cells by spinoculation. HIV-1 infection was determined by measuring GLuc activity 36 hr later. HIV-1 infection by cell-to-cell transmission was assessed by co-culture of HIV-1-infected Jurkat-inGLuc donor cells with target primary CD4+ T cells. Specifically, Jurkat-inGLuc cells were inoculated with HIV-1_NL4-3_, washed, stimulated with 6.25 ng/mL of PMA for 2 hr at 37°C, washed again, and incubated for 18 hr at 37°C. HIV-1_GLuc_-generating donor cells were then washed and co-cultured with target primary CD4+ T cells at a ratio of 1∶1. Infection was determined by measuring GLuc activity 36 hr post-infection.

To test the effectiveness of commonly used antiretroviral inhibitors against both modes of HIV-1 transmission, we established an experimental system that measures cell-free and cell-to-cell transmission with sufficient sensitivity. This system employs a *Gaussia* luciferase (GLuc)-based reporter genome (HIV-1_inGLuc_), which expresses and secretes GLuc only after splicing of an intron (inGLuc), packaging into viral particles and infection of target cells [20], [35]. To test cell-free infection, we inoculated primary CD4+ T cells with HIV-1_NL4-3_ carrying the spliced GLuc reporter (HIV-1_NL4-3-GLuc_) and measured HIV-1 infection 36 hr post-infection (Fig. 1). To measure transmission from donor T cells to primary CD4+ T target cells, we used a Jurkat cell line stably carrying the HIV-1_inGLuc_ reporter (Jurkat-inGLuc). Jurkat-inGLuc cells were transduced with full length HIV-1_NL4-3_ so that donor T cells generated HIV-1_NL4-3-GLuc_ particles and were co-cultured with primary CD4+ T cells (Fig. 1). Although, we used full length HIV-1, the level of infection in primary CD4+ T cells measured at 36 hr post-infection represents a single round of the HIV-1 life cycle (Supplementary Fig. S1A). The incubation period of 36 hr post-infection was selected since we found it to be optimal for the expression and secretion of luciferase (Supplementary Fig. S1B). Under these co-culture conditions, HIV-1 cell-to-cell transmission is 2–3 orders of magnitude more efficient, making the contribution from cell-free spread within the co-culture negligible [20]. To directly compare co-culture infection to cell-free infection, we adjusted the inoculum accordingly so that both modes of transmission resulted in equal percentage of infected target cells (Supplementary Fig. S1C). This ensured that a critical difference between both modes of transmission was the higher number of particles transferred during HIV-1 cell-to-cell transmission while target cells and infection levels remained constant [18], [19], [20]. Sorting of infected target cells, followed by *Alu*-PCR revealed that the average number of integration sites was ∼6-fold higher during HIV-1 cell-to-cell as compared to cell-free transmission (Supplementary Fig. S1D–F). To study the effect of PIs against cell-to-cell transmission, we adjusted the experimental design to account for the activity of this drug class within the donor cell (Supplementary Fig. S3A, Materials and Methods).

### Most NNRTIs, Ent-Is and PIs are effective against HIV-1 cell-to-cell transmission

**Figure 2:**
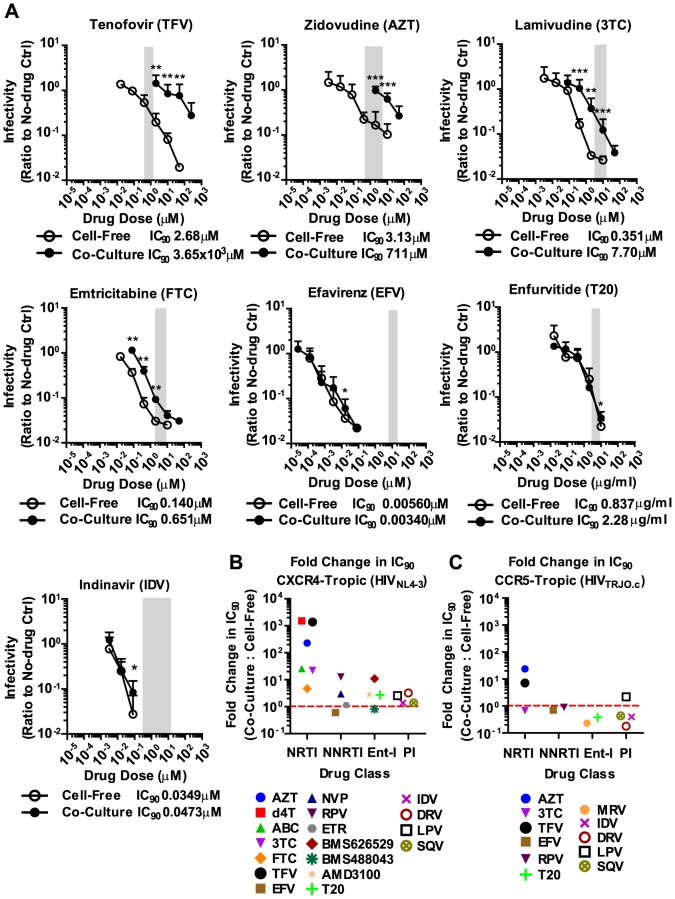
Most NNRTIs Ent-Is and PIs potently inhibit HIV-1 cell-to-cell transmission. (**A**) The efficacy of antiretroviral NRTIs, NNRTIs, Ent-Is and PIs was tested in cell-free or co-culture infection of primary CD4+ T cells. The data is displayed as a double log plot of the ratio of the relative GLuc light units measured for the inhibitor over the DMSO-only control versus increasing inhibitor concentration. Plateau signals above and below the effective drug concentrations are excluded from the figure and from analysis. The gray area represents the drug concentrations detected in the serum of treated patients (C_ave_ to C_Max_ for NRTIs and C_Min_ to C_Max_ for other drugs). Error bars represent the standard deviation from the combination of at least two individual experiments each done in triplicate. Asterisks indicate statistical significance of the difference between HIV-1 cell-to-cell transmission and cell-free infection at each drug concentration. It was calculated using the Mann-Whitney U test: * p≤0.05, ** p≤0.01, *** p≤0.001. The IC_90_ calculated for cell-free or co-culture infection is given beneath each graph. (**B**) The change in IC_90_ for co-culture over cell-free infection was plotted for each drug and grouped based on drug family. The dashed red line indicates no change. The complete drug inhibition curves for stavudine (d4T), abacavir (ABC), rilpivirine (RPV), nevirapine (NVP), etravirine (ETR), plerixafor (AMD3100), enfurvitide (T20), the indicated BMS inhibitors, indinavir (IDV), darunavir (DRV), lopinavir (LPV), and saquinavir (SQV) are shown in Supplementary Fig. S2 and S3. (**C**) The efficacy of a subset of antiretroviral inhibitors was tested in cell-free or co-culture infection of primary CD4+ T cells with the founder virus clone pTRJO.c [37]. All curves are shown in Supplementary Fig. S5.

We applied these experimental conditions to systematically test the efficacy of 6 NRTIs, 4 NNRTIs, 4 Ent-Is and 4 PIs against cell-free and cell-to-cell HIV-1 transmission. The NRTI inhibitors TFV, AZT, and stavudine (d4T) were profoundly impaired in their ability to interfere with HIV-1 cell-to-cell transmission to primary human CD4+ T cells (Fig. 2A, Supplementary Fig. S2). Their dose-response curves were right-shifted indicating that ∼200–1000-fold higher drug concentrations were required to interfere with HIV-1 cell-to-cell transmission as compared to cell-free HIV-1. This observation is consistent with previous observations for TFV and AZT [32], [34] and translates into poor HIV-1 inhibition at the active drug concentrations detected in the serum of treated patients (Fig. 2A, gray bar). Interestingly, the NRTI inhibitors lamivudine (3TC), abacavir (ABC) and emtricitabine (FTC) showed a narrowing of cell-free and cell-to-cell transmission dose-response curves indicating an increased ability to interfere with HIV-1 cell-to-cell transmission relative to other NRTIs (Fig. 2A and Supplementary Fig. S2). Importantly, most NNRTIs (nevirapine (NVP), etravirine (ETR) and efavirenz (EFV)) interfered with HIV-1 cell-to-cell transmission as efficiently as with cell-free transmission. The Ent-Is enfurvitide (T20), plerixafor (AMD3100), and BMS488043 were also very effective consistent with previous results for T20 [30]. Rilpivirine (RPV) and BMS626529 exhibited intermediate effects (Supplementary Fig. S2). The PIs indinavir (IDV), darunavir (DRV), lopinavir (LPV) and saquinavir (SQV) also retained their effectiveness regardless of the mode of transmission (Fig. 2A and Supplementary Fig. S3B), consistent with recent observations [36]. The effectiveness of most NNRTIs, Ent-Is and PIs is clearly visible when the fold change in the IC_90_ during cell-to-cell transmission versus cell-free HIV-1 transmission is plotted for each drug (Fig. 2B). The effects could not be attributed to drug toxicity (Supplementary Fig. S4). A similar pattern was observed for a more physiologically relevant founder virus HIV-1_TROJ.c_ (Fig. 2C and Supplementary Fig. S5) [37]. Cell-to-cell transmission of HIV-1_TROJ.c_ was more resistant to TFV and AZT, albeit to a lesser extent than HIV-1_NL4-3_, and remained highly sensitive to NNRTIs, Ent-Is and PIs.

**Figure 3:**
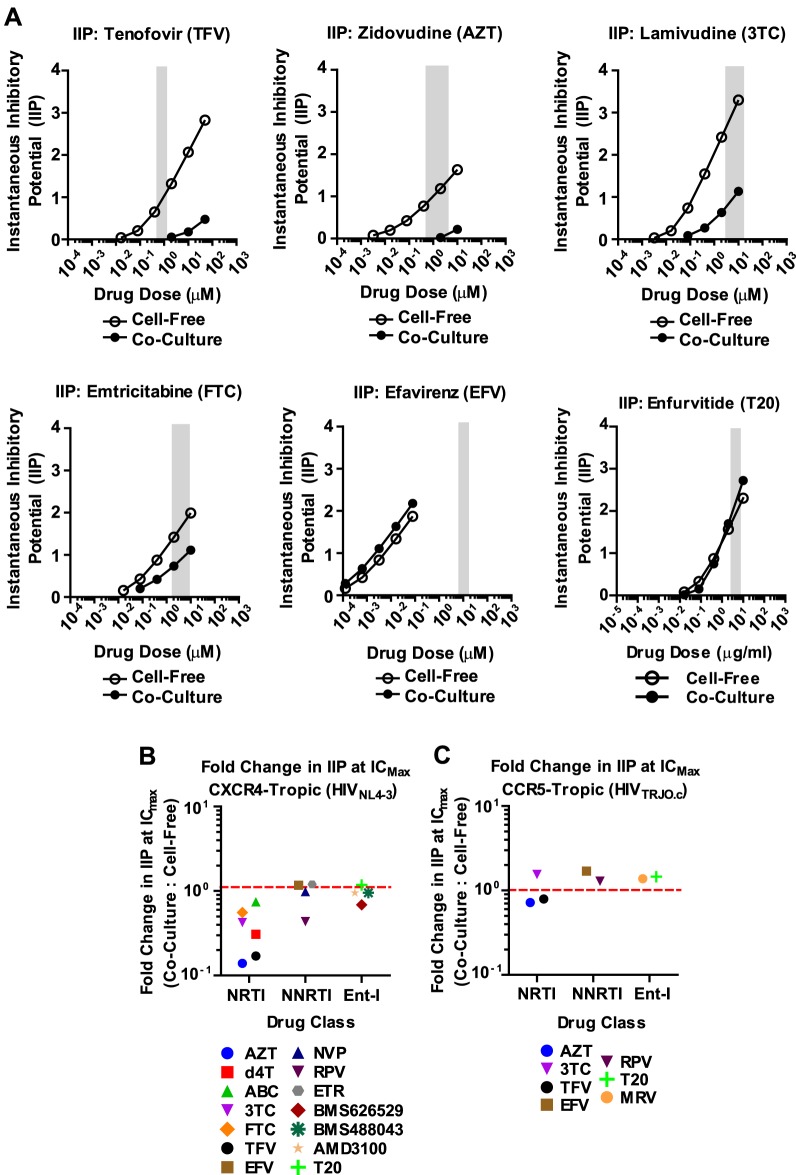
Most NNRTI and Ent-Is maintain their instantaneous inhibitory potential against HIV-1 cell-to-cell transmission. (**A**) The average instantaneous inhibitory potential (IIP) was calculated for each drug of Fig. 2A and Supplementary Fig. S2. All curves are shown in Supplementary Fig. S6. (**B**) The change in average IIP at the top inhibitor dose (IC_Max_) for co-culture over cell-free infection was plotted for each drug and grouped based on drug family. (**C**) The average IIP was calculated for the drugs tested against HIV-1_TRJO.c_ and the change in the average IIP at IC_Max_ was grouped based on drug family. All IIP curves are shown in Supplementary Fig. S7.

To gain a better understanding of the effectiveness of antiretroviral inhibitors in both modes of HIV-1 transmission, we calculated the instantaneous inhibitory potential (IIP) [38], [39]. The IIP incorporates both the IC_50_ and the slope of the inhibition curve and may provide a more accurate assessment of the effectiveness of an inhibitor. We found that the IIP in co-culture samples was dramatically weakened for TFV and AZT and significantly reduced for most other NRTIs (Fig. 3A, B and Supplementary Fig. S6). Importantly, the IIP was not affected for most NNRTIs and Ent-Is in agreement with the observations based on IC_90_. All data is summarized as the ratio of the IIP at the top drug dose (IC_Max_) for co-culture over cell-free in Figures 3B and C. All curves are shown in Supplementary Fig. S6 and S7. The IIP could not be computed for PIs because of the limited dynamic range in the signal for HIV-1 cell-to-cell transmission (data now shown). These data demonstrate that while some antiretroviral drugs such as NRTIs are less efficient against HIV-1 cell-to-cell transmission, most NNRTIs and Ent-Is remain highly effective regardless of the mode of viral transmission.

### Combination therapies are highly effective against HIV-1 cell-to-cell transmission

**Figure 4:**
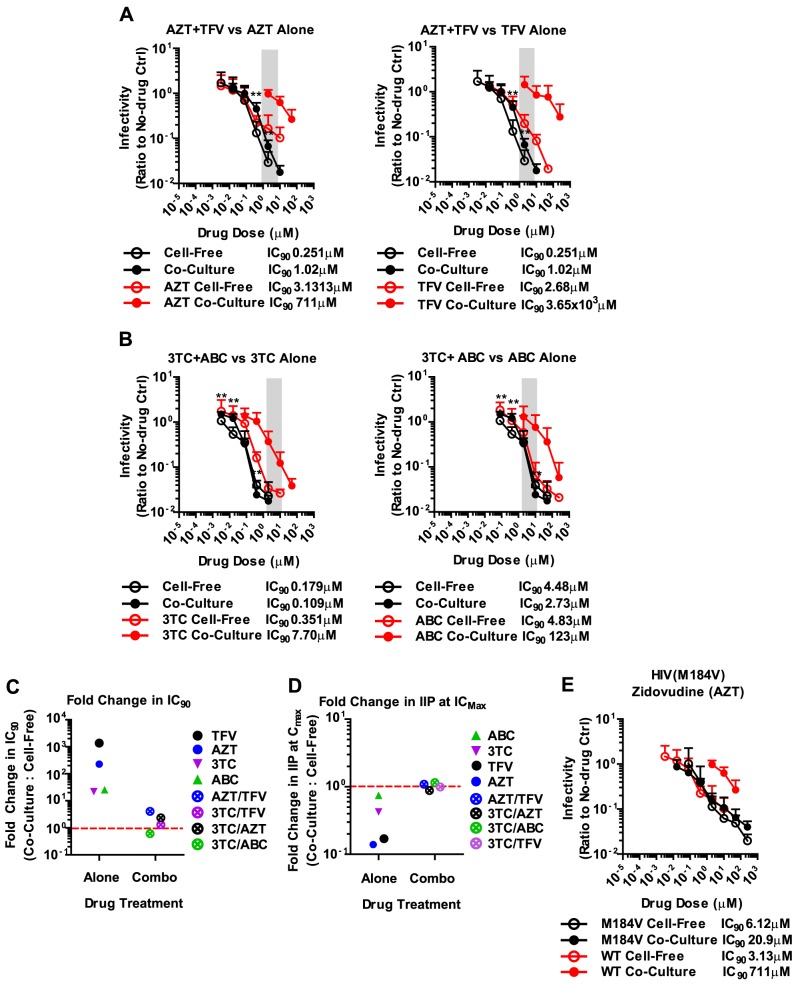
Combinations of NRTIs are highly effective against HIV-1 cell-to-cell transmission. (**A, B**) An experiment as in Fig. 2 was performed for the combinations (black lines) of (**A**) AZT and TFV and (**B**) 3TC and ABC and compared to each single inhibitor treatment (red lines, left and right panel). The X-axis represents the drug concentration of the drug within the combination that is being compared to the single drug treatment (in red). See Supplementary Fig. S8A for additional inhibitor combinations. (**C**) The change in IC_90_ for co-culture over cell-free infection for the single inhibitors was compared to all the inhibitor combinations tested. (**D**) The change in average IIP at IC_Max_ for co-culture over cell-free infection for the single inhibitors was compared to all the inhibitor combinations tested. See Supplementary Fig. S8B for complete set of average IIP data. (**E**) Cell-free and co-culture infection of primary cells with HIV-1_NL4-3_ carrying the M184V mutation of reverse transcriptase (black line) compared to wild-type HIV-1_NL4-3_ (red line) in the presence of increasing concentrations of AZT. Error bars represent the standard deviation from the combination of at least two individual experiments each done in triplicate.

The failure of antiretroviral inhibitors such as TFV and AZT to interfere with HIV-1 cell-to-cell transmission stands in conflict with the clinical experience that they are effective in suppressing HIV-1 replication in AIDS patients [1], [2], [3], [4]. However, mono-therapy is not used for the treatment HIV-1-infected patients due to the high risk of emergence of drug-resistant mutants [40], [41]. Thus, we wondered whether drugs that fail to interfere with cell-to-cell transmission when used individually, are more effective when used in combination. To test drug combinations, we matched drug concentrations according to their IC_90_ values and treated co-culture and cell-free infections with serially diluted drug combinations. Strikingly, the combination of AZT and TFV potently interfered with HIV-1 cell-to-cell transmission (Fig. 4A). While each drug individually was ∼200–1000-fold less effective against HIV-1 cell-to-cell transmission, this difference was reduced to ∼4.1-fold when the drugs were combined (Fig. 4A). Furthermore, the drug combination shifted the effective dose-range required to suppress HIV-1 cell-to-cell transmission to within the drug concentrations detected in the serum of treated AIDS patients (Fig. 4A, gray bar). This observation was reproduced for three additional combinations of NRTIs including the clinically used combinations of 3TC/ABC and 3TC/AZT (Fig. 4B, Supplementary Fig. S8A) [42]. The increased effectiveness of combination therapy was also visible when the IC_90_ values were compared and the IIP was calculated (Fig. 4C, D and Supplementary Fig. S8B).

The effectiveness of combination therapies was surprising since drug combinations at most doubled the total drug concentration. If the effectiveness of competitive NRTI inhibitors was reduced due to a high MOI at sites of cell-cell contact [32], then doubling the drug concentration should be insufficient to inhibit all the incoming particles (Fig. 2). The observation of synergy in NRTI combination therapies can likely be explained by more efficient inhibition of reverse transcriptase. During reverse transcription, reverse transcriptase is able to excise an incorporated nucleotide analog, thus lowering the potential effectiveness of many NRTIs [43], [44], [45]. Combinations of nucleotide analogs have been observed to interfere with this excision process, thus enhancing the ability of NRTIs to terminate the growing DNA chain [46]. To test this hypothesis, we conducted our co-culture and cell-free inoculations using an HIV-1_NL4-3_ clone carrying the M184V mutation in RT. This mutation renders HIV-1 reverse transcriptase hypersensitive to AZT due to its inability to excise the drug [47], [48]. We predicted that AZT would efficiently interfere with HIV-1 cell-to-cell transmission of HIV-1 carrying M184V mutant RT. Indeed, the difference in IC_90_ between cell-free and co-culture infection was dramatically reduced compared to HIV-1 carrying wild-type RT (Fig. 4E). These results suggest that synergy between NRTIs against HIV-1 cell-to-cell transmission is, at least in part, due to a reduction of NRTI excision, which in turn causes more efficient chain termination.

Next, we asked how this drug-resistant HIV-1 mutant would behave during combination therapies in both modes of transmission. The M184V mutation was first characterized as a mutation that provides resistance against 3TC [49], [50]. We hypothesized that if this mutant were to be exposed to a combination of 3TC and TFV, it may be able to resist inhibition by TFV by cell-to-cell transmission. We found that if HIV-1 is resistant to one of the inhibitors used in the combination, the dose-response curve for cell-to-cell transmission was shifted again towards higher drug concentrations, phenocopying the behavior of NRTI mono-therapy (Supplementary Fig. S9). This suggests that drug-resistant HIV-1 mutants may gain a replicative advantage to amplify by cell-to-cell transmission in the presence of some combination therapies.

### Antiretroviral inhibitors and combinations that are effective against HIV-1 cell-to-cell transmission are also effective against high viral MOI

**Figure 5:**
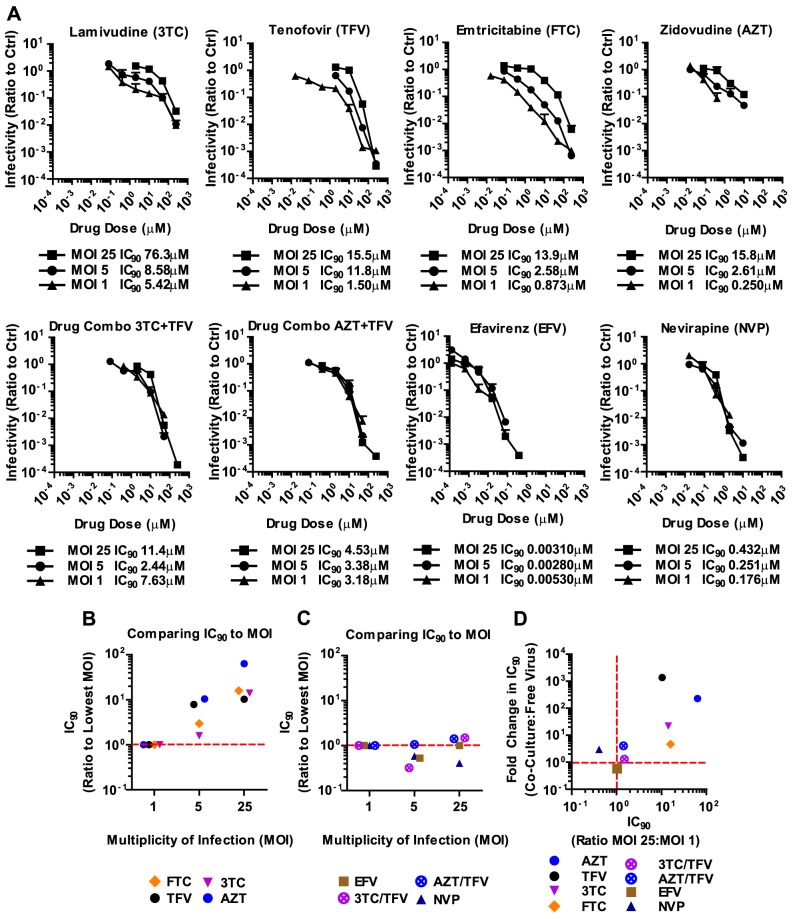
Antiretroviral inhibitors and combinations that are effective against HIV-1 cell-to-cell transmission are effective against high viral MOI. (**A**) An experiment as in Fig. 2 was performed for cell-free HIV-1 infection of MT-4 cells with increasing MOI in the presence of indicated single or combined antiretroviral inhibitors. Error bars represent the standard deviation of 3 measurements. (**B**) Changes in IC_90_ relative to MOI = 1 were plotted for each viral dose to display the MOI-dependence of NRTIs. (**C**) Changes in IC_90_ relative to MOI = 1 were plotted for increasing viral MOI to display the MOI-independence of NNRTIs and inhibitor combinations. (**D**) Graph displays the correlation between the effectiveness of ART inhibitors and combinations against HIV-1 cell-to-cell transmission and MOI-independence. Specifically, the comparison of the change in IC_90_ of co-culture over cell-free infection is plotted over the change in IC_90_ of MOI = 25 over MOI = 1.

It has been suggested that the high local MOI observed at sites of cell-cell contact is responsible for the relative resistance of HIV-1 cell-to-cell transmission to antiretroviral inhibitors [32], [33]. This would suggest that the reason why most NNRTIs and all combination therapies are effective against HIV-1 cell-to-cell transmission is because they are MOI-independent, thus would remain effective despite high viral MOI. To test this hypothesis, we concentrated HIV-1_NL4-3(GLuc)_ and used highly susceptible MT4 cells, which allowed us to use MOIs of up to 25. An MOI of 25 is close to the highest MOI that can be detected during HIV-1 cell-to-cell transmission [18], [19], [20]. We found that 3TC, TFV, FTC and AZT were indeed overpowered by increasing particle numbers (Fig. 5A, B). In other words, higher drug concentrations were required for these NRTIs to inhibit high MOIs. In striking contrast, NNRTIs and combination therapies were largely MOI-independent (Fig. 5A, C). The same drug concentration of NVP or the combination of AZT and TFV inhibited HIV-1 irrespective of the MOI. The strong correlation between non-effectiveness or effectiveness of ART against HIV-1 cell-to-cell transmission and high MOI was best seen when the change in IC_90_ during co-culture infection was plotted versus the change in IC_90_ during high MOI (Fig. 5D). This plot shows the clustering of MOI-dependent and MOI-independent treatments. Thus, we predict that those individual and combination therapies that are effective against high MOI will also efficiently interfere with HIV-1 cell-to-cell transmission.

## Discussion

The recent questioning of ART's effectiveness during HIV-1 cell-to-cell transmission [32] stood in conflict with the clinical experience that HAART is effective at suppressing HIV-1 replication in patients [1], [2], [3], [4]. Many clinicians may have concluded that HIV-1 cell-to-cell transmission cannot be relevant in patients and that cell-free spread must dominate. Here we showed that this interpretation is likely incorrect. Rather, we demonstrate that clinically applied ART regimens are effective against HIV-1 cell-to-cell transmission likely because they also remain effective against the high number of particles transferred at sites of cell-cell contacts. By systematically testing the efficacy of commonly used antiretroviral inhibitors against cell-to-cell and cell-free HIV-1 transmission, we demonstrate that while some NRTIs are indeed less effective against HIV-1 cell-to-cell transmission, most NNRTIs, Ent-Is and PIs remain highly effective. Importantly, upon combining of 2 NRTIs that failed as single therapies, HIV-1 cell-to-cell transmission and cell-free infection often became equally inhibited. Therefore, our findings indicate that the ability of HIV-1 cell-to-cell transmission to evade antiretroviral drug inhibition is not a universal phenomenon. Because standard treatment involves the combination of several drugs (2 NRTI+1 NNRTI or PI) it would seem unlikely that HIV-1 cell-to-cell transmission would provide a feasible mechanism for any ongoing viral replication in the presence of suppressive treatment. This observation is consistent with a large body of evidence indicating that suppressive HAART stops any measurable level of viral replication [51].

Our observations that combination therapies of NRTIs can be effective against HIV-1 cell-to-cell transmission indicates that the clinical effectiveness of HAART did not automatically imply that HIV-1 spreads by cell-free virus in patients. Rather we demonstrate that HAART effectively suppresses the high MOI observed during HIV-1 cell-to-cell transmission. The determination of the exact mechanism of HIV-1 cell-to-cell spread *in vivo* will require the direct *in vivo* visualization of viral dissemination [52], [53]. However, our results already provide evidence that HIV-1 cell-to-cell transmission can contribute to the pathogenesis of HIV-1 as a feasible mechanism of viral escape during drug mono-therapy or inadequate treatment regimens. We confirmed the original observation that some NRTIs fail to restrict HIV-1 cell-to-cell transmission during mono-therapy [32]. We also provide evidence that drug-resistant virus may gain a replicative advantage to spread by HIV-1 cell-to-cell transmission in the presence of inadequate combination therapy. Thus, HIV-1 cell-to-cell transmission may contribute to the rise of drug-resistant virus and therapy failure under conditions of poor adherence [54].

Our finding that ART similarly suppresses high viral MOIs and HIV-1 cell-to-cell transmission is consistent with the suggestion that a high viral MOI is a central feature associated with cell-cell contact mediated viral dissemination [18], [19], [20], [32]. High MOIs have been observed in infected cells in tissues *in vivo* [21], [22]. This observation appears to be in conflict with the finding that most circulating T cell lymphocytes carry only a single provirus [27], [28]. However, a high MOI may often result in bystander death of CD4+ lymphocytes, a hallmark of AIDS pathogenesis [23]. Primary cells have been suggested to innately sense the presence of a large number of viral DNA copies (unintegrated and/or integrated) and undergo apoptosis and/or pyroptosis [24], [25], [26]. The cell death of highly infected cells may result in the positive selection of CD4+ T cells that carry a single provirus [27], [28]. The ability of ART to suppress the high viral MOI documented in this report confirms the long standing knowledge that effective ART is able to effectively suppress bystander cell death and protect most AIDS patients from further T cell depletion [4], [55].

A high local MOI of reverse transcriptase can overwhelm drug activity by mass action [32], [33]. However, the ability of multiple drugs, particularly NNRTIs, to remain effective against the high local MOI observed during HIV-1 cell-to-cell transmission suggests that mass action alone cannot fully explain the mechanism by which antiretroviral inhibitors function under these conditions. In the case of NRTIs, our data suggest that the ability of reverse transcriptase to excise nucleotide analogs plays an important role in this phenomenon. When nucleotide excision was inhibited through mutation of the RT, mono-therapy with a nucleotide analog can inhibit both modes of viral transmission with similar efficiency. Similarly, we observed synergy in combination therapies consistent with more efficient reverse transcript chain termination and less efficient nucleotide analogue excision by RT [46]. In the case of NNRTIs, allosteric inhibition of RT also provides for synergistic effects [46]. Moreover, we hypothesize that other steps in the cellular uptake, metabolism, or secondary binding sites, determine the effective dosage of antiretroviral inhibitors. Said differently, under conditions of high MOI encountered during cell-to-cell transmission, interaction of the drug with RT is not the rate-limiting step for efficient inhibition of reverse transcription. That is, the number of incoming RT molecules alone does not define the effective dosages of drug. These considerations indicate that there is likely no single mechanism that explains whether a drug or drug combination is effective against HIV-1 cell-to-cell transmission. Thus, each drug and drug combination needs to be tested.

To this day, therapy outcome in patients has been difficult to predict. Mathematical models have been developed recently that incorporate drug IC_50_, and the slopes of inhibition curves as in the IIP, as well as viral fitness, mutations and treatment adherence [56], [57]. Our data indicate that the effectiveness of ART against HIV-1 cell-to-cell transmission and viral MOI are additional helpful parameters to predict drug efficacy. Moreover, we observed that all drugs effective against HIV-1 cell-to-cell transmission were effective because they are MOI-independent and can efficiently suppress the high local MOI at virological synapses. These data suggest that highly effective drug regimens, either single or in combination therapies, must exhibit MOI-independence. Testing the effectiveness of antiretroviral inhibitors against increasing MOI provides a simple assay and a valuable tool for screening existing and novel individual drugs and combination therapies prior to clinical testing.

## Materials and Methods

### Ethics statement

All the cells used in this study were anonymized and were obtained from commercially available sources (ATCC, AIDS Research and Reagents Program, New York Blood Center). As such, these samples are exempt from IRB review.

### Cells

Peripheral blood mononuclear cells were purified from blood enriched by leukapheresis (New York Blood Center) with the Ficoll-Paque Plus gradient (GE Healthcare Life Sciences). Following this purification step, CD4+ T cells were purified using the EasySep Human CD4+ T Cell Enrichment Kit (StemCell Technologies) and were stimulated with PHA (10 µg/mL) (Sigma-Aldrich), IL-2 (100 U/mL), and IL-7 (100 ng/mL) for 72 hr (cytokines from Miltenyi Biotec) at 37°C. After stimulation, cells were maintained in RPMI (Gibco) supplemented with 100 U/mL penicillin/streptomycin (Gibco), 2 mM of L-glutamine (Gibco), 10% FBS (Gibco), IL-2 (100 U/mL), and IL-7 (100 ng/mL) at 37°C. A subclone of Jurkat-inGLuc was selected from the population described by Zhong, et al. [20]. The cell lines Jurkat-inGLuc, MT4 (NIH AIDS Research and Reagents Program), and HEK293 (ATCC) were maintained in RPMI supplemented with 100 U/mL penicillin/streptomycin, 2 mM of L-glutamine, and 10% FBS at 37°C. TZMbl cells were obtained from the NIH Research and Reagents Program and were maintained in DMEM supplemented with 100 U/mL penicillin/streptomycin, 2 mM of L-glutamine, and 10% FBS at 37°C.

### Plasmids

The plasmid encoding the intron-regulated HIV-based *Gaussia* luciferase pUCHR-inGLuc (HIV_inGLuc_) was kindly donated by Gisela Heidecker, National Cancer Institute. The plasmid encoding the HIV-1 molecular clones NL4-3 [58] and pTRJO.c [37] were obtained from the AIDS Research and Reagents Program. The plasmid encoding the M184V mutation in reverse transcriptase (pNL4-3ΔEnv(M184V)) was kindly donated by Robert Siliciano, Johns Hopkins University. To generate a wild type version of the M184V mutant, the construct was digested with PspOMI and AgeI (New England Biolabs). The ∼1.5 kb fragment generated was then ligated to the ∼13 kb fragment of wild type NL4-3 after digestion with the same enzymes. The plasmid encoding the vesicular stomatitis virus G-glycoprotein (VSV-G) was obtained from Michael Marks, University of Pennsylvania.

### Reagents

Most antiretroviral drugs tested in this study were obtained from the AIDS Research and Reagents Program. The attachment inhibitors BMS488043 and BMS626529 were donated by Mark Krystal (Bristol-Myers Squibb) [59], [60], [61].

### Viruses

HIV-1 pseudotyped with VSV-G was generated by co-transfecting HEK293 cells with pVSV-G and pNL4-3 or pTRJO.c at a ratio of 1∶10. HIV_GLuc_ was generated by co-transfecting HEK293 cells with pNL4-3 (or pTRJO.c) and pHIV_inGLuc_ at a ratio of 6∶1 or 10∶1. For inoculations of MT4 cells, HIV_GLuc_ was generated by inoculating HEK293 cells stably carrying HIV_inGLuc_ and collecting culture supernatant at 36 and 60 hr post-infection. Viral supernatants were concentrated using Lenti-X Concentrator (Clontech) or by ultracentrifugation (∼20,000×g) over a 20% sucrose (in PBS) cushion for 2 hr at 4°C.

### Cell-free and co-culture experiments

Primary CD4+ T cells were incubated with serial dilutions of nucleoside analogs at 37°C for 16–24 hr prior to inoculation in a total of 1% DMSO. This is required for the accumulation of sufficient concentrations of active inhibitors within the cells. Cells were incubated at 37°C with non-nucleoside analogs and entry inhibitors for 2 hr prior to inoculation also in a total of 1% DMSO. Cell-free inoculations were conducted by spinoculating 10^5^ primary CD4+ T cells in 96-well plates at 1,200×g and at room temperature for 2 hr with 50 µL of concentrated HIV_GLuc_ [62]. Cultures were then incubated at 37°C for 36–40 hr.

Co-cultures were conducted by first spinoculating Jurkat-inGLuc cells with full length HIV-1_NL4-3_ pseudotyped with VSV-G at 1,200×g and at room temperature for 2 hr. The Jurkat-inGLuc clone was originally selected to be CD4-low cells to minimize donor-to-donor infection in co-culture experiments with target primary CD4+ T cells. Cells were then washed, stimulated with 6.25 ng/mL of PMA for 2 hr at 37°C, washed and incubated in fresh medium for 18 hr at 37°C. A brief PMA treatment was used to stimulate expression of latent HIV_in-GLuc_ for efficient packaging by the incoming wild type HIV. Additionally, PMA treatment causes down-regulation of CD4 expression in the donor Jurkat-inGLuc cells, further preventing donor-to-donor infection [63]. Subsequently, PMA was removed from the culture so that target primary CD4+ T cells were never exposed to the drug. 10^5^ infected Jurkat-inGLuc cells were then washed and co-cultured with 10^5^ primary CD4+ T cells in a total of 50 µL. GLuc accumulated in the culture supernatant was detected using the BioLux *Gaussia* Luciferase Assay Kit (New England Biolabs) and a Berthold Technologies luminometer.

To test PIs, this protocol had to be modified to account for the activity of this drug class within the HIV-1 donor cell. To do this, HIV-1 infected Jurkat-inGLuc cells were incubated with increasing concentrations of PIs immediately following stimulation with PMA for 12 hr prior to co-culturing with primary cells (see Supplementary Fig. S3A). Co-cultures were incubated for 42 hr prior to measuring GLuc. To assess the effect of protease inhibitors on the infectivity of cell-free particles, we collected the supernatant of donor cells cultured alone in the presence of PIs 54 hr after exposure to the inhibitors. This supernatant corresponds to the total number of particles released during the co-culture. The supernatant was tittered on 10^5^ target primary CD4+ T cells or on 2×10^4^ TZMbl target cells at a total volume of 60 µL in 96-well plates, spinoculated and incubated at 37°C for 36 hr prior to measuring GLuc activity. TZMbl cells were used to assess the infectivity of the supernatant because they are much more susceptible to cell-free HIV-1_NL4-3_ than primary CD4 T cells and could detect very low titers of HIV-1_NL4-3_ produced by donor cells.

### Flow cytometry

Prior to infection, target cells were stained with 1 µM of Cell Proliferation Dye eFluor 670 (eBioscience) in OptiMEM medium (Gibco) at 37°C for 20 min. Cells were washed and incubated in complete medium supplemented with cytokines at 37°C for 30 min, washed and prepared for drug treatment. 24 hr after infection, cultures were harvested and fixed in 100 µL of BD CytoFix/CytoPerm buffer (BD Biosciences) for at least 30 min at 4°C. The cells were then washed with BD Perm/Wash buffer (BD Biosciences) and stained for 30 min at 4°C in 100 µL of BD Perm/Wash buffer containing the anti-HIV-1 Gag antibody clone KC57 (Beckman Coulter). The cells were washed with BD Perm/Wash buffer, resuspended in PBS supplemented with 0.5% BSA and 2 mM of EDTA and analyzed by flow cytometry with a FACSCalibur (BD Biosciences). The same staining protocol was used for sorting HIV-1-positive target cells after cell-free or cell-to-cell transmission. The sort was conducted using a BD FACSAria sorter.

### Measuring HIV-1 integration from sorted samples

Following the sort, cells were spun, resuspended in 200 µL of PBS +200 µL of Buffer AL (Qiagen) +20 µL of Proteinase K (Qiagen) and incubated at 60°C for 24 h to remove paraformaldehyde. DNA was purified using the DNeasy Blood and Tissue Kit (Qiagen). HIV-1 integration was measured by *Alu*-PCR as previously described using 2.5 U of Platinum Taq (Life Technologies) [64].

### Cell viability

36 hr post-infection, a sample of 10 µL of culture was collected for each drug treatment condition and mixed with 10 µL of CellTiter-Glo (Promega). Cells were incubated at 37°C for 10 min and the luciferase signal was measured using a Berthold Technologies luminometer.

### Data analysis and statistics

Inhibitor IC_90_ and IIP were calculated using MATLAB software. Statistical tests were calculated using Minitab software.

## Supporting Information

Figure S1 **Kinetics of cell-free and cell-to-cell transmission of HIV-1.** (**A**) Kinetics of HIV-1 infection in cell-free and co-culture system measured after stopping the progress of infection at selected time points using a combination of efavirenz (1 µM) and saquinavir (1 µM) and measuring GLuc activity at 48 hr post-infection. The data are displayed as relative GLuc light units over the signal at t = 0 hr. Note that the signal measured 48 hr post-infection represents infection events that took place within the first 12 hr post-infection and thus represents a single round of infection. Error bars represent the standard deviation of the combination of 2–3 experiments each done in triplicate. (**B**) Kinetics of GLuc expression in cell-free and co-culture system followed by measuring GLuc expression at the indicated time points. The data are displayed as the GLuc signal ratio over the signal at t = 0 hr. These results indicate that 36–48 hr are required for an optimal level of signal. Error bars represent the standard deviation of the combination of 2–3 experiments each done in triplicate. (**C**) Cell-free and co-culture infections were adjusted to result in ∼10% infection of target cells. Percent infection was determined based on flow cytometry analysis of HIV-1 Gag expression at 24 hr post-infection. Error bars represent the standard deviation of 10 measurements from 5 experiments. (**D**) Primary CD4+ T cells were infected by cell-free inoculation or co-culture infection as in panel (A) and the infected population of primary CD4+ T cells was sorted 36 hr after infection in order to determine the actual viral MOI resulting from either mechanism of viral transmission. Sorting gates were placed based on an efavirenz-treated control (1 µM). The purity of the sorted population is shown. (**E**) GLuc signals obtained after cell-free or co-culture infection. (**F**) The viral MOI was determined by measuring HIV-1 integration by *Alu*-PCR. The level of integration in efavirenz-treated samples was undetectable (<10^−4^ copies/cell, not shown).
(PDF)

Figure S2 **Most NNRTIs and Ent-Is potently inhibit HIV-1_NL4-3_ cell-to-cell transmission.** Complete data set for inhibitors presented in Fig. 2. The serum drug concentration range for BMS626529 and BMS488043 are based on C_Min_ to C_Max_ of the best trial conditions described by Nettles, *et al.* and Hanna, *et al.* respectively [60], [65].
(PDF)

Figure S3 **Most PIs potently inhibit HIV-1_NL4-3_ cell-to-cell transmission.** (**A**) Experimental outline for testing PIs against HIV-1 cell-to-cell transmission. Briefly, Jurkat-inGLuc cells were inoculated with HIV-1_NL4-3_, washed, stimulated with PMA, washed and cultured in the presence of increasing concentrations of PIs. One set of cells was incubated for 12 hr at 37°C prior to co-culturing with target primary CD4+ T cells. Co-cultures were incubated for 42 hr followed by measuring GLuc. The other set of cells was incubated without target cells for 54 hr at 37°C. This corresponds to the cell-free virus generated and released by donor cells. The viral supernatant was tittered on target primary CD4+ T cells or TZMbl cells and measured GLuc signal 36 hr later. (**B**) Inhibition curves for the data shown in Fig. 2B. Error bars represent the standard deviation from the combination of at least two individual experiments each done in triplicate.
(PDF)

Figure S4 **Treatment with antiretroviral inhibitors does not cause a significant effect on the viability of the primary CD4+ T cells.** Viability of cells infected by cell-free HIV-1_NL4-3_ or co-culture at 36 hr post-infection determined with the CellTiter-Glo kit. The data are displayed as the percent viability compared to DMSO control. Error bars represent the standard deviation for 3 measurements.
(PDF)

Figure S5 **Most NNRTIs, Ent-Is, and PIs potently inhibit HIV-1_TRJO.c_ cell-to-cell transmission.** Tested the effect of selected antiretroviral inhibitors against cell-free and cell-to-cell transmission of the founder virus HIV-1_TRJO.c_. (**A**) The percentage of infected target cells was equivalent regardless of the mode of transmission. (**B**) Inhibition curves for the data shown in Fig. 2C. Cell-free virus signal for samples treated with PIs was measured by titrating virus produced from donor cells on primary CD4+ T cells. (**C**) Viability of cells after co-culture or cell-free infection. Error bars represent the standard deviation from the combination of at least two individual experiments each done in triplicate.
(PDF)

Figure S6 **Most NNRTIs and Ent-Is keep a high instantaneous inhibitory potential (IIP) against HIV-1_NL4-3_ cell-to-cell transmission.** Complete IIP data set for inhibitors presented in Fig. 3A, B.
(PDF)

Figure S7 **Most NNRTIs and entry inhibitors keep a high instantaneous inhibitory potential (IIP) against HIV-1_TRJO.c_ cell-to-cell transmission.** Complete IIP for the HIV-1_TRJO.c_ data set presented in Fig. 3C.
(PDF)

Figure S8 **Combinations of NRTIs are highly effective against HIV-1 cell-to-cell transmission.** (**A**) An experiment as in Fig. 4A, B for HIV-1_NL4-3_ was performed for the combinations of 3TC with TFV and 3TC with AZT (**B**) The average IIP for all drug combinations presented in Fig. 4 was compared to the average IIP of single inhibitor treatment. Error bars represent the standard deviation from the combination of at least two individual experiments each done in triplicate.
(PDF)

Figure S9 **Drug resistant HIV-1 gains an advantage to spread by cell-to-cell transmission in the presence of drug combinations.** (**A**) An experiment as in Fig. 2 for HIV-1_NL4-3_ carrying the M184V mutation of reverse transcriptase (black line) compared to wild-type HIV-1_NL4-3_ (red line) in the presence of increasing concentrations of the 3TC with TFV drug combination. Error bars represent the standard deviation from the combination of 4–5 experiments.
(PDF)
