# Supplementary figures and images for: Highly Active Antiretroviral Therapies Are Effective against HIV-1 Cell-to-Cell Transmission
Source: PLoS Pathog. 2014 Feb 27;10(2):e1003982. doi: 10.1371/journal.ppat.1003982 (PMC3937346; doi:10.1371/journal.ppat.1003982)

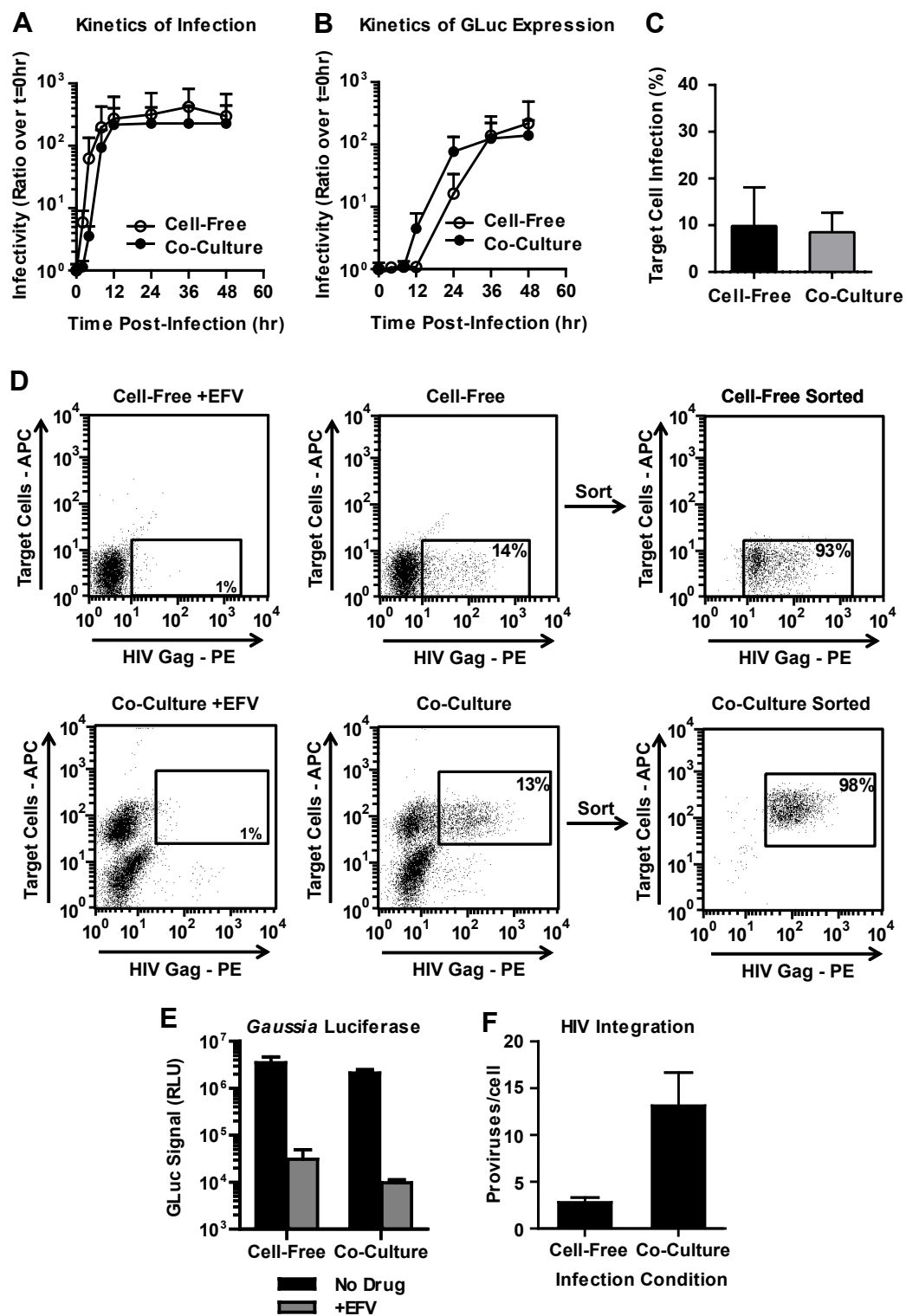

Supplementary Figure S1.

Supplement: Figure S1 — Kinetics of cell-free and cell-to-cell transmission of HIV-1. (A) Kinetics of HIV-1 infection in cell-free and co-culture system measured after stopping the progress of infection at selected time points using a combination of efavirenz (1 µM) and saquinavir (1 µM) and measuring GLuc activity at 48 hr post-infection. The data are displayed as relative GLuc light units over the signal at t = 0 hr. Note that the signal measured 48 hr post-infection represents infection events that took place within the first 12 hr post-infection and thus represents a single round of infection. Error bars represent the standard deviation of the combination of 2–3 experiments each done in triplicate. (B) Kinetics of GLuc expression in cell-free and co-culture system followed by measuring GLuc expression at the indicated time points. The data are displayed as the GLuc signal ratio over the signal at t = 0 hr. These results indicate that 36–48 hr are required for an optimal level of signal. Error bars represent the standard deviation of the combination of 2–3 experiments each done in triplicate. (C) Cell-free and co-culture infections were adjusted to result in ∼10% infection of target cells. Percent infection was determined based on flow cytometry analysis of HIV-1 Gag expression at 24 hr post-infection. Error bars represent the standard deviation of 10 measurements from 5 experiments. (D) Primary CD4+ T cells were infected by cell-free inoculation or co-culture infection as in panel (A) and the infected population of primary CD4+ T cells was sorted 36 hr after infection in order to determine the actual viral MOI resulting from either mechanism of viral transmission. Sorting gates were placed based on an efavirenz-treated control (1 µM). The purity of the sorted population is shown. (E) GLuc signals obtained after cell-free or co-culture infection. (F) The viral MOI was determined by measuring HIV-1 integration by Alu-PCR. The level of integration in efavirenz-treated samples was undetect [file ppat.1003982.s001.pdf]

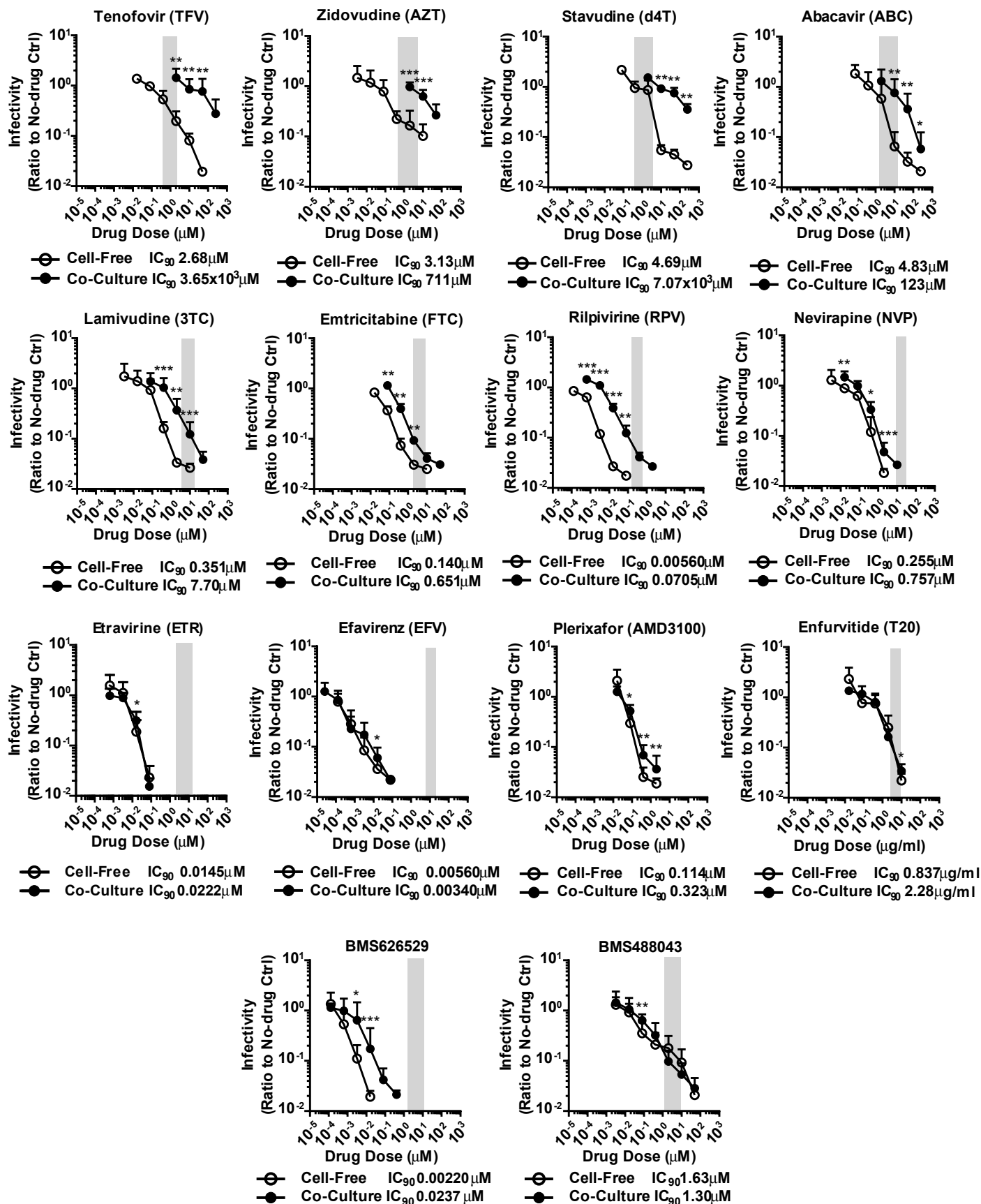

Supplementary Figure S2.

Supplement: Figure S2 — Most NNRTIs and Ent-Is potently inhibit HIV-1NL4-3 cell-to-cell transmission. Complete data set for inhibitors presented in Fig. 2. The serum drug concentration range for BMS626529 and BMS488043 are based on CMin to CMax of the best trial conditions described by Nettles, et al. and Hanna, et al. respectively [60], [65]. (PDF) [file ppat.1003982.s002.pdf]

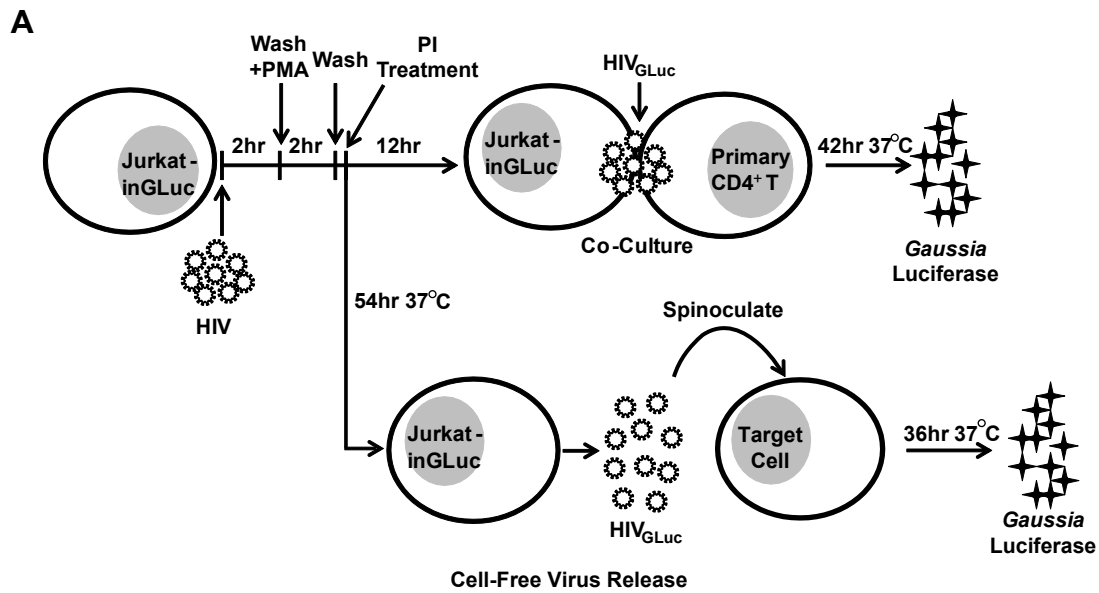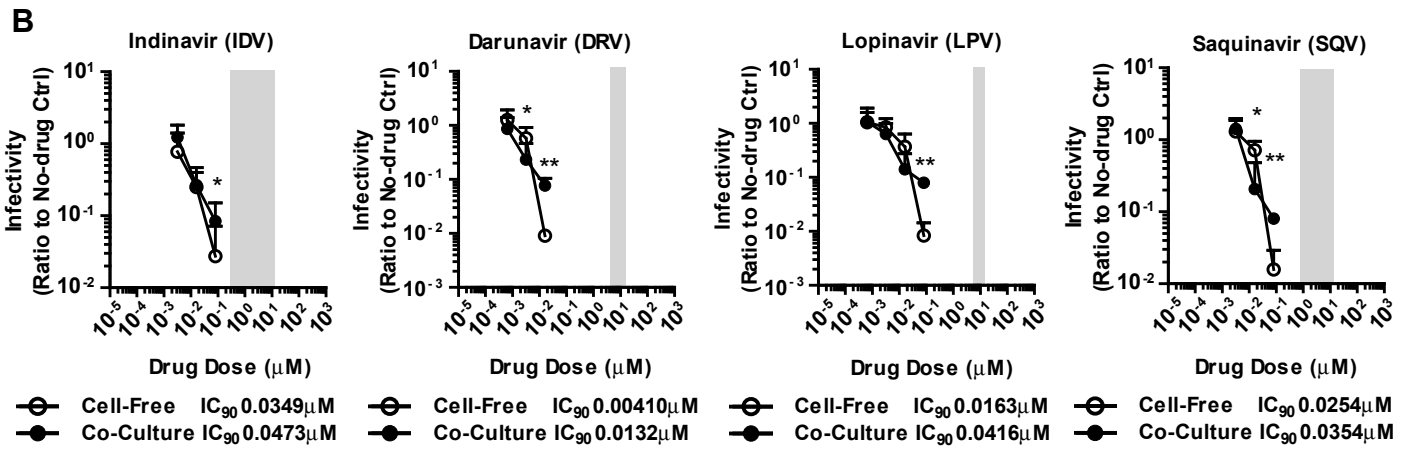

Supplementary Fig. S3

Supplement: Figure S3 — Most PIs potently inhibit HIV-1NL4-3 cell-to-cell transmission. (A) Experimental outline for testing PIs against HIV-1 cell-to-cell transmission. Briefly, Jurkat-inGLuc cells were inoculated with HIV-1NL4-3, washed, stimulated with PMA, washed and cultured in the presence of increasing concentrations of PIs. One set of cells was incubated for 12 hr at 37°C prior to co-culturing with target primary CD4+ T cells. Co-cultures were incubated for 42 hr followed by measuring GLuc. The other set of cells was incubated without target cells for 54 hr at 37°C. This corresponds to the cell-free virus generated and released by donor cells. The viral supernatant was tittered on target primary CD4+ T cells or TZMbl cells and measured GLuc signal 36 hr later. (B) Inhibition curves for the data shown in Fig. 2B. Error bars represent the standard deviation from the combination of at least two individual experiments each done in triplicate. (PDF) [file ppat.1003982.s003.pdf]

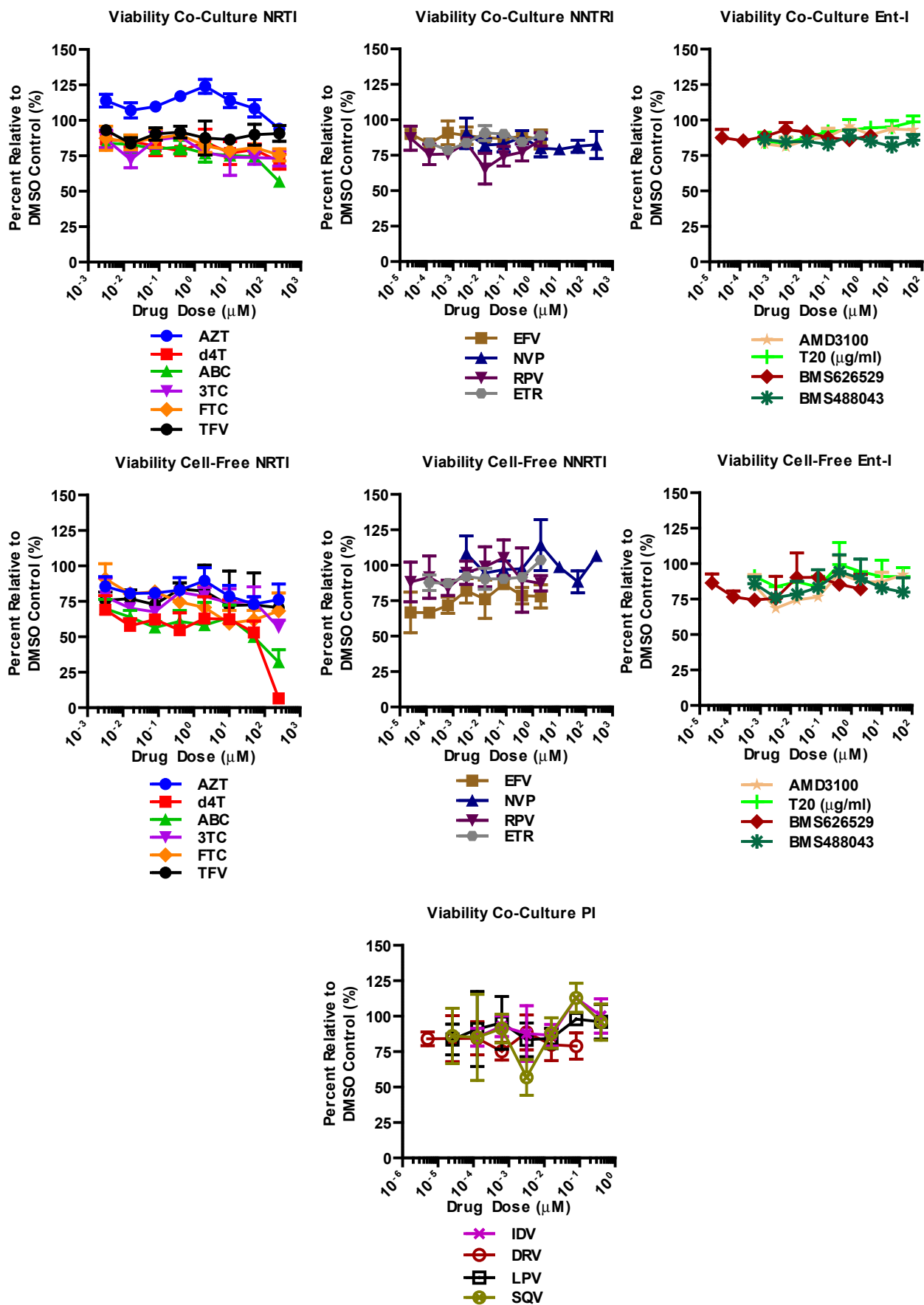

Supplementary Figure S4.

Supplement: Figure S4 — Treatment with antiretroviral inhibitors does not cause a significant effect on the viability of the primary CD4+ T cells. Viability of cells infected by cell-free HIV-1NL4-3 or co-culture at 36 hr post-infection determined with the CellTiter-Glo kit. The data are displayed as the percent viability compared to DMSO control. Error bars represent the standard deviation for 3 measurements. (PDF) [file ppat.1003982.s004.pdf]

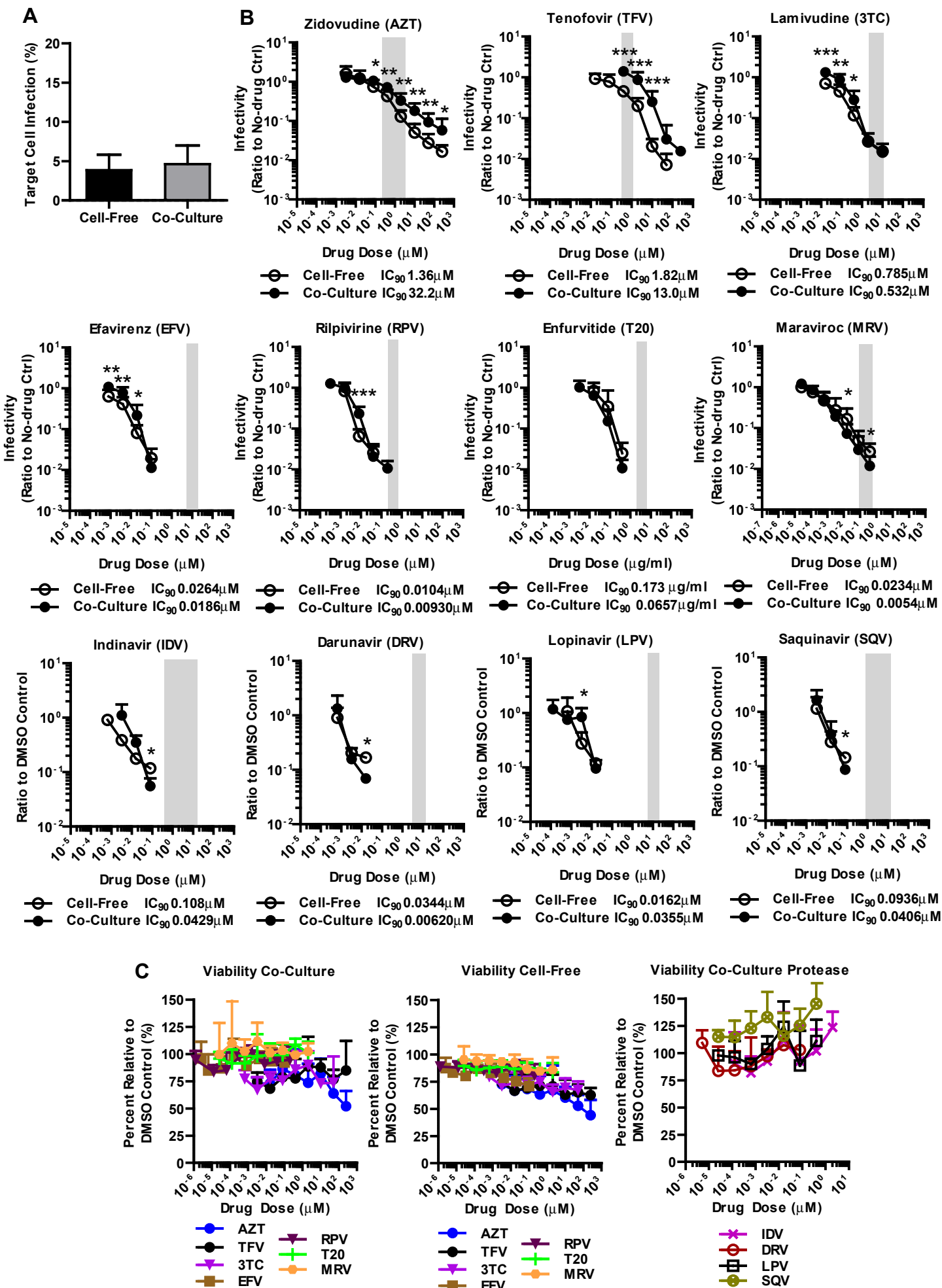

Supplementary Figure S5.

Supplement: Figure S5 — Most NNRTIs, Ent-Is, and PIs potently inhibit HIV-1TRJO.c cell-to-cell transmission. Tested the effect of selected antiretroviral inhibitors against cell-free and cell-to-cell transmission of the founder virus HIV-1TRJO.c. (A) The percentage of infected target cells was equivalent regardless of the mode of transmission. (B) Inhibition curves for the data shown in Fig. 2C. Cell-free virus signal for samples treated with PIs was measured by titrating virus produced from donor cells on primary CD4+ T cells. (C) Viability of cells after co-culture or cell-free infection. Error bars represent the standard deviation from the combination of at least two individual experiments each done in triplicate. (PDF) [file ppat.1003982.s005.pdf]

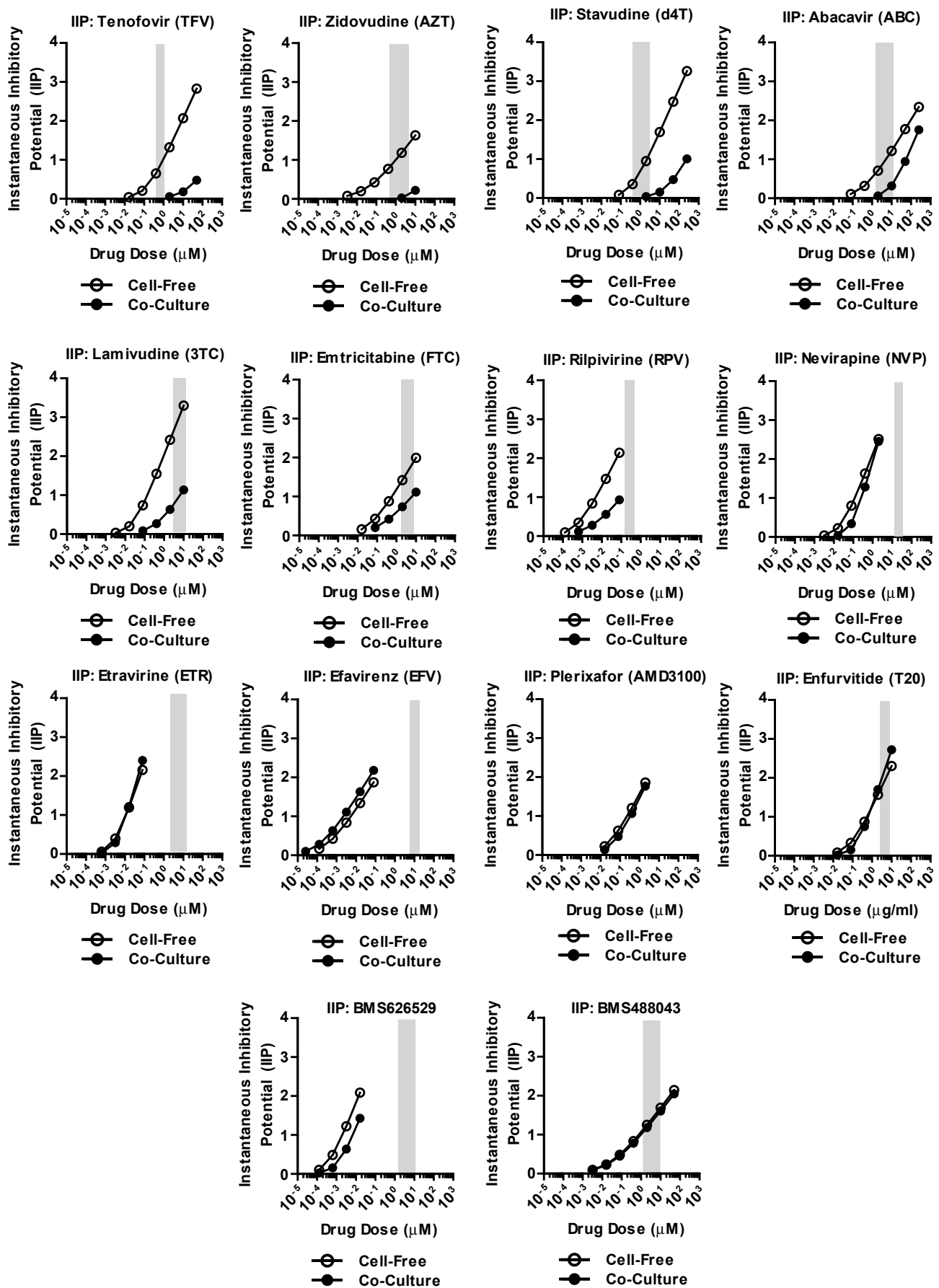

Supplementary Figure S6.

Supplement: Figure S6 — Most NNRTIs and Ent-Is keep a high instantaneous inhibitory potential (IIP) against HIV-1NL4-3 cell-to-cell transmission. Complete IIP data set for inhibitors presented in Fig. 3A, B. (PDF) [file ppat.1003982.s006.pdf]

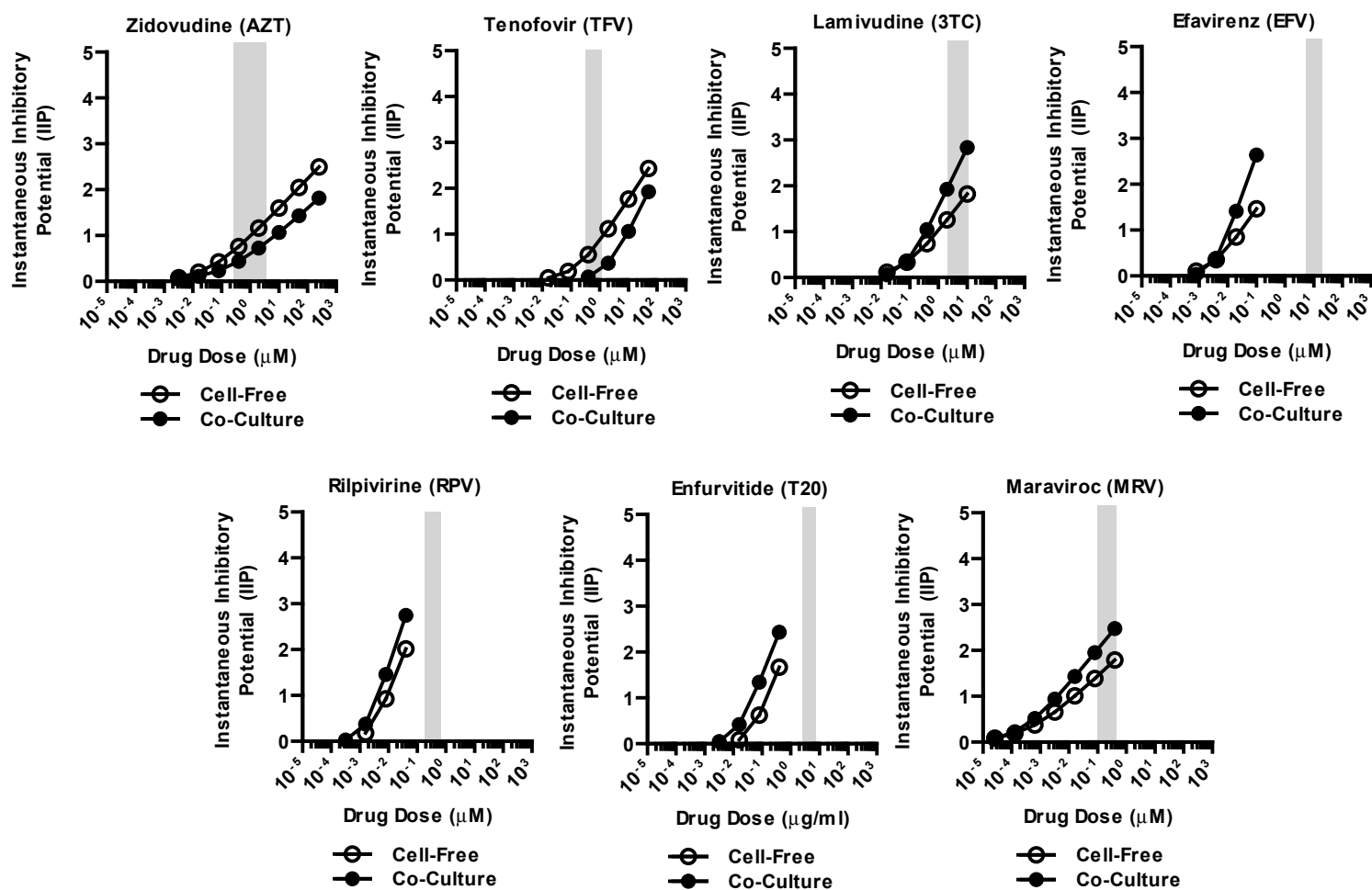

Supplementary Figure S7.

Supplement: Figure S7 — Most NNRTIs and entry inhibitors keep a high instantaneous inhibitory potential (IIP) against HIV-1TRJO.c cell-to-cell transmission. Complete IIP for the HIV-1TRJO.c data set presented in Fig. 3C. (PDF) [file ppat.1003982.s007.pdf]

**A**

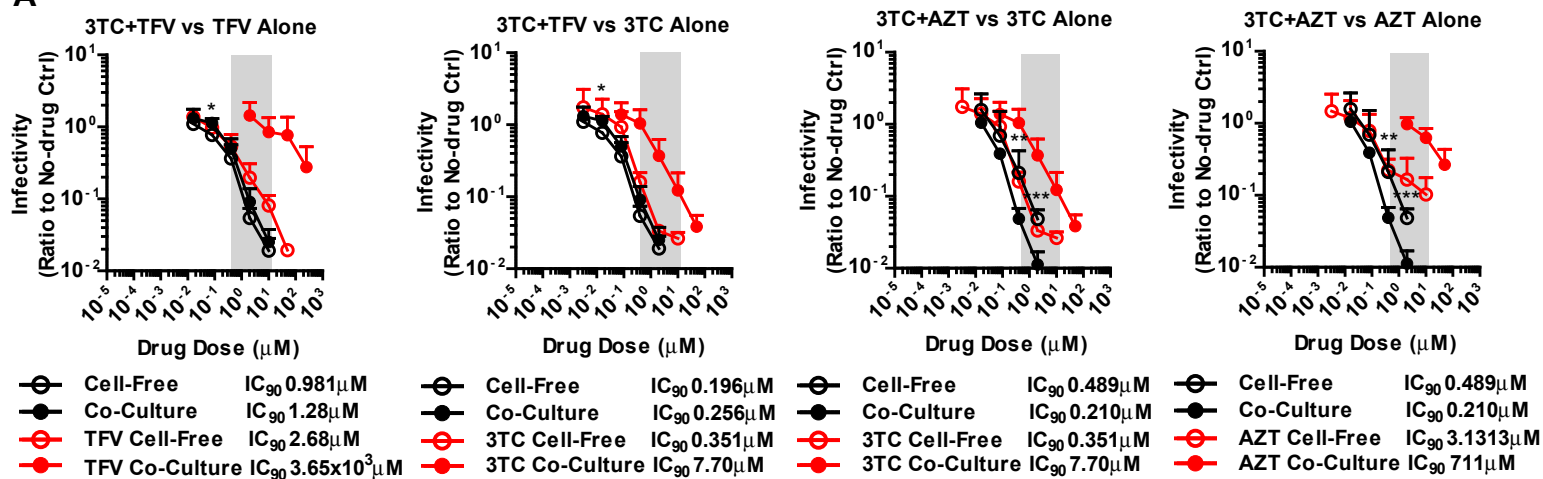

**B**

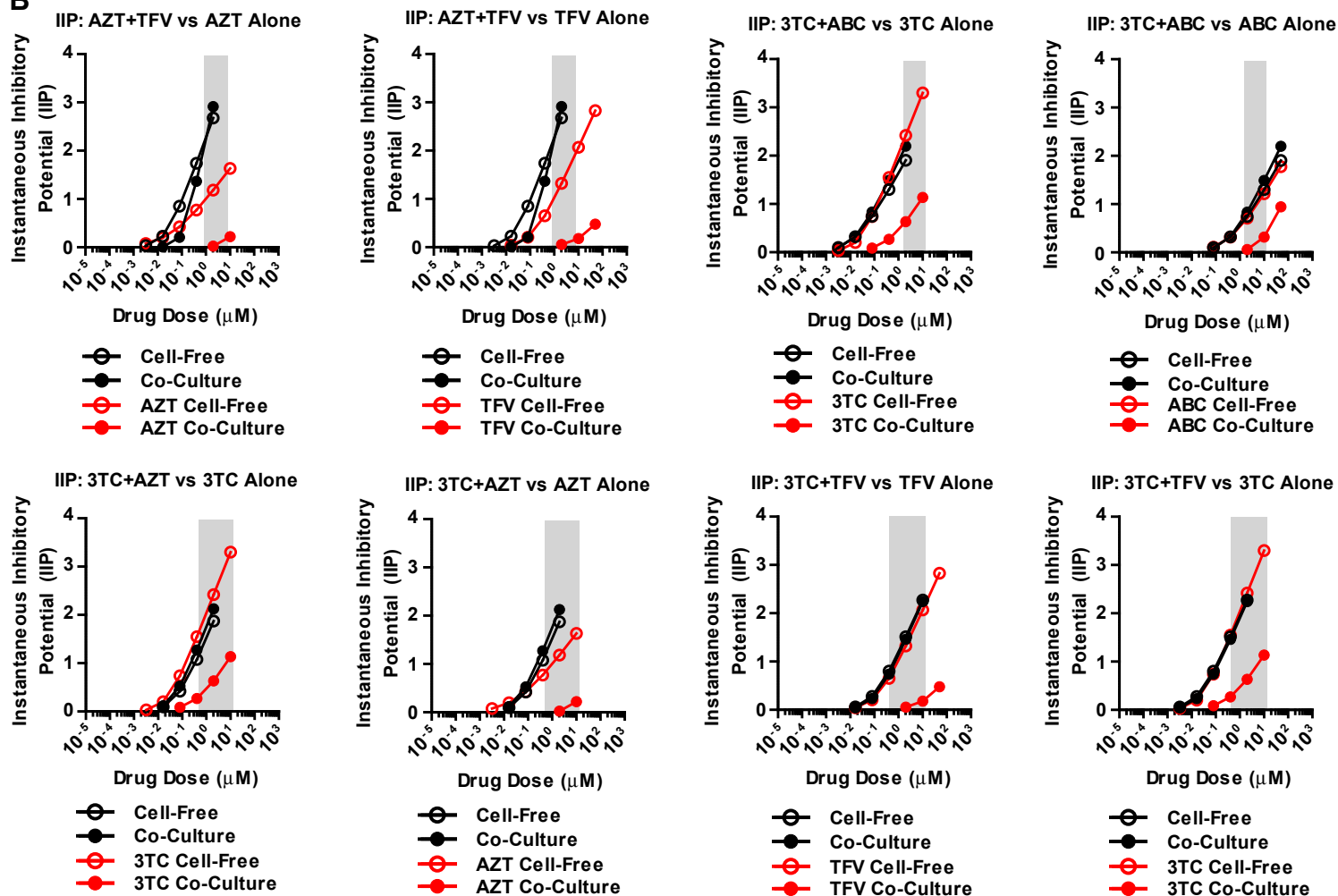

Supplementary Figure S8.

Supplement: Figure S8 — Combinations of NRTIs are highly effective against HIV-1 cell-to-cell transmission. (A) An experiment as in Fig. 4A, B for HIV-1NL4-3 was performed for the combinations of 3TC with TFV and 3TC with AZT (B) The average IIP for all drug combinations presented in Fig. 4 was compared to the average IIP of single inhibitor treatment. Error bars represent the standard deviation from the combination of at least two individual experiments each done in triplicate. (PDF) [file ppat.1003982.s008.pdf]

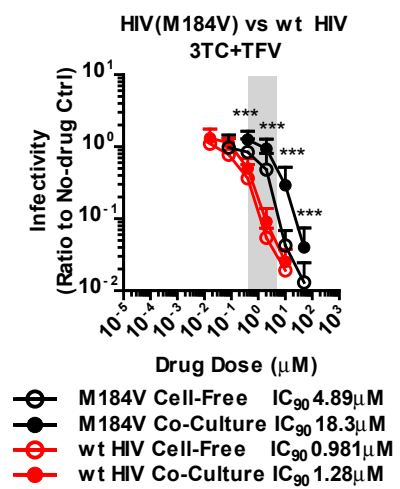

Supplementary Figure S9.

Supplement: Figure S9 — Drug resistant HIV-1 gains an advantage to spread by cell-to-cell transmission in the presence of drug combinations. (A) An experiment as in Fig. 2 for HIV-1NL4-3 carrying the M184V mutation of reverse transcriptase (black line) compared to wild-type HIV-1NL4-3 (red line) in the presence of increasing concentrations of the 3TC with TFV drug combination. Error bars represent the standard deviation from the combination of 4–5 experiments. (PDF) [file ppat.1003982.s009.pdf]
